# In Vitro Study to Assess Effective Cleaning Techniques for Removing *Staphylococcus aureus* from Menstrual Cups

**DOI:** 10.3390/ijerph19031450

**Published:** 2022-01-27

**Authors:** Nadine Wunsch, Stefan J. Green, Sebastian Adam, Janie Hampton, Penelope A. Phillips-Howard, Supriya D. Mehta

**Affiliations:** 1 Sustainability Team, Einhorn Cups, 10997 Berlin, Germany; nadine@einhorn.my; 2 Department of Internal Medicine and Genomics and Microbiome Core Facility, Rush University, Chicago, IL 60612, USA; Stefan_Green@rush.edu; 3 Hycoma Microbiology Research Laboratory, Biofilm Management, 48149 Muenster, Germany; s.adam@hycoma.de; 4 Independent Researcher, Oxford OX4 2EZ, UK; janie@janiehampton.co.uk; 5 Department of Clinical Sciences, Liverpool School of Tropical Medicine, Liverpool L3 5QA, UK; Penelope.Phillips-Howard@lstmed.ac.uk; 6 Division of Epidemiology & Biostatistics, University of Illinois Chicago School of Public Health, Chicago, IL 60612, USA

**Keywords:** menstrual cups, menstrual cup cleaning, menstrual management, menstruation, *Staphylococcus aureus*

## Abstract

Background: We sought to determine the effectiveness of common cleaning procedures in eliminating *S. aureus* from silicone menstrual cups. Methods: In this in vitro study, we tested four cleaning techniques: (1) cold water; (2) cold water and liquid soap; (3) cold water followed by steeping the cup in boiled water for 5 min in a ceramic mug covered with a small plate; and (4) cold water and soap followed by steeping the cup in boiled water as in (3). Human blood was coated to the inner and outer surface of each cup, dried, and incubated with 10^6^ *S. aureus* colony-forming units (CFU/mL). All tests were performed in triplicate. Viable bacterial abundance was measured with decadic dilution and drop plate or surface plating. Results: Bacteria were most effectively eliminated by cleaning cups with soap and water and then steeping in boiled water (0 CFU/cup vs. 2.075 × 10^8^/cup no cleaning, *p* = 0.005). This was not statistically significantly different from washing cups with water only and steeping 5 min in boiled water (14 CFU/cup). Raised lettering on the outer surface of the menstrual cups resulted in more bacterial recovery from pieces with lettering than without lettering. Conclusions: These results advance knowledge of between-period menstrual cup cleaning recommendations, suggesting that the logistical challenges of continuous boiling may be eliminated with steeping at least 5 min.

## 1. Introduction

Healthy management of menstruation requires access to water, sanitation, puberty and hygiene health education, both for individuals and communities, as well as effective, safe and affordable menstrual products with access to disposal facilities. Accessibility and affordability are particularly relevant in impoverished areas, where the lack of resources affects work and school attendance and productivity during menstruation. Demand for environmentally friendly products that produce less waste is increasing [1,2,3]. The re-usable menstrual cup is a convenient, comfortable, safe, economic and eco-friendly choice for menstruation [4,5,6,7]. The menstrual cup is inserted into the vagina below the cervix, where it collects rather than absorbs menstrual flow. Menstrual cups can hold 10–38 mL of blood and, depending on the menstrual flow, are emptied every 4–12 h, and cleaned instead of being thrown away after a single use [4]. Menstrual cups are usually made of medical-grade silicone, while a few brands use thermoplastic elastomer (TPA) or rubber [5].

Toxic shock syndrome (TSS) was first identified in 1978 and in the early 1980s, when cases of menstrual TSS (mTSS) were reported, associated with tampons. mTSS is caused by the production of the toxin TSST-1 by *Staphylococcus aureus* [8]. TSST-1 is only produced at a neutral pH in the vagina and in the presence of oxygen. mTSS is also called the ‘tampon disease’ since the air trapped within tampons may facilitate the aerobic production of TSST-1 by *S. aureus* in the typically anaerobic vaginal environment [9]. The composition of tampons had changed from cotton to more absorbent viscose, which encouraged users to leave them in for longer [10]. As with other internal period products, menstrual cups may carry a risk of menstrual toxic shock syndrome (mTSS). However, in a systematic review in 2019, there were only five reported cases of mTSS in connection with the usage of menstrual cups [5], among tens of millions of menstrual cup users [11]. 

There is concern that a buildup of menstrual effluent creates a microbial biofilm, which could increase the risk of mTSS [12]. Thus, menstrual cup users need to ensure that biofilm formation and maturation are minimized to keep the risk for TSS as low as possible. Different brands recommend a variety of cleaning techniques, ranging from solely wiping the cup to boiling it [13], though most brands recommend boiling their cup for up to 10 min. This conflicting information suggests that rigorous testing of the most appropriate and practical way to clean menstrual cups is needed for the continued safe use of these products at a global scale. Furthermore, in low-income and middle-income countries (LMIC), some users may not have easy access to space or materials to boil the cup as recommended [14,15,16]. 

The goal of this study is to compare different cleaning techniques for their effectiveness in removing biofilms of *S. aureus* from the surface of menstrual cups. The study aims to address the knowledge gap related to appropriate cleaning of menstrual cups and to provide consumers of these cups information on the best cleaning practice. Specifically, the effectiveness of various cleaning procedures in eliminating pathogenic, biofilm forming bacteria from menstrual cups was assessed using the einhorn brand menstrual cups and four different cleaning methods.

## 2. Materials and Methods

This study was determined as exempt from human subject ethical review by the University of Illinois Chicago Institutional Review Board.

### 2.1. Experimental Design/Material

*S. aureus* DSM 25630 from Jeonom University Hospital (South Korea) was used (Leibniz Institute, DSMZ German Collection of Microorganisms and Cell Cultures GmbH, Braunschweig, Germany). This strain can produce the toxic shock syndrome toxin 1 (TSST-1), is resistant to multiple antibiotics, including methicillin, and is considered a methicillin resistant *S. aureus* (MRSA). The strain contains a gene for enterotoxin G and has been typed as Spa type t002. According to the DSMZ (German Collection of Microorganisms and Cell Cultures GmbH), the strain grows optimally at a human body temperature of 37 °C. 

**Figure 1:**
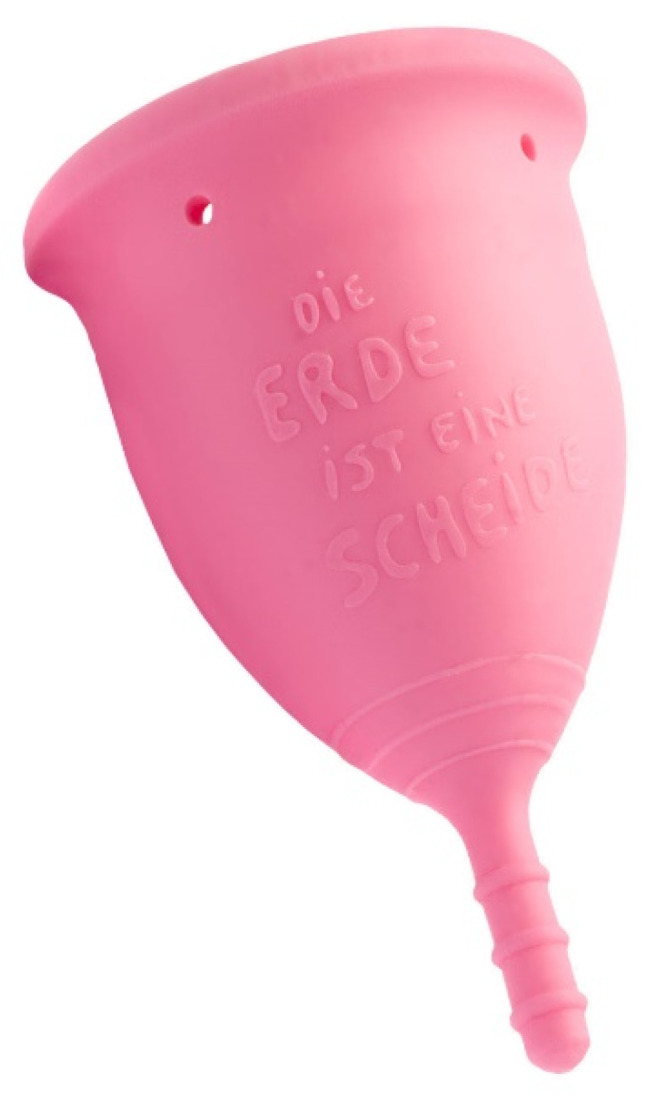
Small einhorn menstrual cup.

The material tested was the “Papperlacup” einhorn brand menstrual cup [17]. There are two cup sizes: small holds up to 17 mL of blood (Figure 1) and medium holds up to 26 mL of blood, which was used for the whole cup tests. These cups are made from 100% medical-grade silicone. The silicone is manufactured by WACKER (Burghausen, Germany), and the cups are produced by Divine (Kippenheim, Germany).

Biofilm development on the different surfaces of the Papperlacup was analyzed. The surface of the inside of the cup is shiny and smooth, while the outside is matte and contains raised lettering, as can be seen in Figure 1. This lettering, present in relief on the external surface, provides additional surface area for biofilm formation.

### 2.2. Cleaning Techniques

This study was conducted to test a range of cleaning techniques on their ability to remove biofilms formed by *S. aureus*. The tested cleaning techniques reflect between-period cleaning instructions commonly included on menstrual cup products as well as real-life cleaning as practiced by menstrual cup users. We tested four cleaning techniques, including cleaning with (1) cold water; (2) cold water and liquid soap (Balea Cremeseife Sensitiv); (3) cold water followed by steeping the menstrual cup in boiled water (250 mL) for 5 min (poured immediately after reaching boiling) in a mug covered with a small plate; and (4) cold water and soap followed by steeping the menstrual cup in boiled water (250 mL) for 5 min (poured immediately after reaching boiling) in a mug covered with a small plate. The room temperature in the lab where experiments were conducted was maintained at 25 °C ± 1 °C. For all four cleaning methods, the cups were rubbed and turned between fingers under running water for 30 s without using a brush or scrub. 

**Figure 2:**
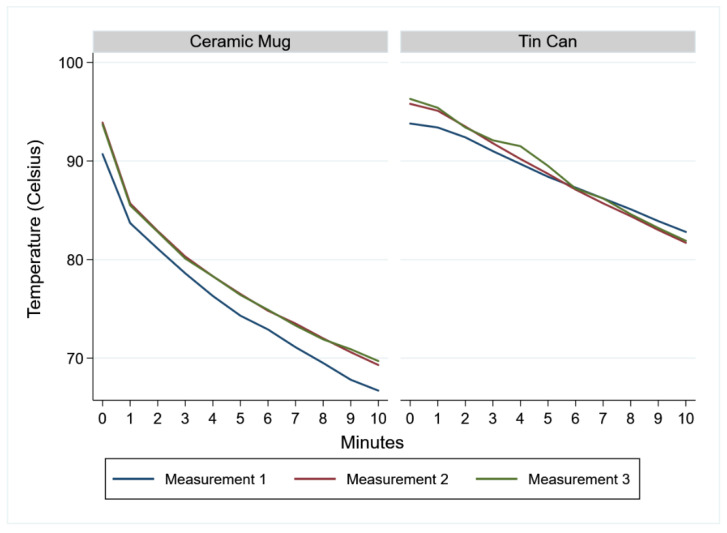
Temperature curves. Legend: Repeat measures of temperature of 250 mL of boiled water in a ceramic mug or tin can left to steep for 10 min. Both the ceramic mug and tin can were kept covered with a saucer.

In our study, a ceramic mug was used for steeping the cups in the boiled water. A new mug was used for each individual cup and cup piece test. As tin cans are more commonly available in low-resource settings, we performed an initial comparison of mugs and tin cans to observe the temperature profiles and to assess whether the mugs could approximate conditions in tin cans. Water was boiled before pouring into mugs and tin cans and measured with a thermometer (Model P300W, TFA Dostmann, Wertheim, Germany). Temperature decline curves were plotted over 10 min (Figure 2), and these demonstrated that heat retention was higher for tin cans. For method comparisons, less than 10-min incubations and ceramic mugs were used to approximate the non-fully adherent sterilization procedures by users. 

### 2.3. Whole Cup Incubations with Staphylococcus aureus 

For complete cup assays, human blood from a lab worker was applied to the entire inner and outer surface of each menstrual cup using a sterile cotton swab (Carlroth). The coated cups were dried in an incubator at 37 °C ± 1 °C for a minimum of 30 min (Memmert^®^ incubator). Control cups without blood were similarly incubated. Subsequently, each cup was placed in 125 mL tryptic soy broth (TSB) medium (CASO-Bouillon) containing *S. aureus* (10^6^ colony forming units (CFU)/mL) or TSB without *S. aureus*. The cups in the TSB medium were incubated for 12 h at 37 °C to match the general maximum recommended wearing time for such cups [13,18]. All tests were performed in experimental triplicate.

### 2.4. Viable Bacterial Cell Abundance Measurement

**Figure 3:**
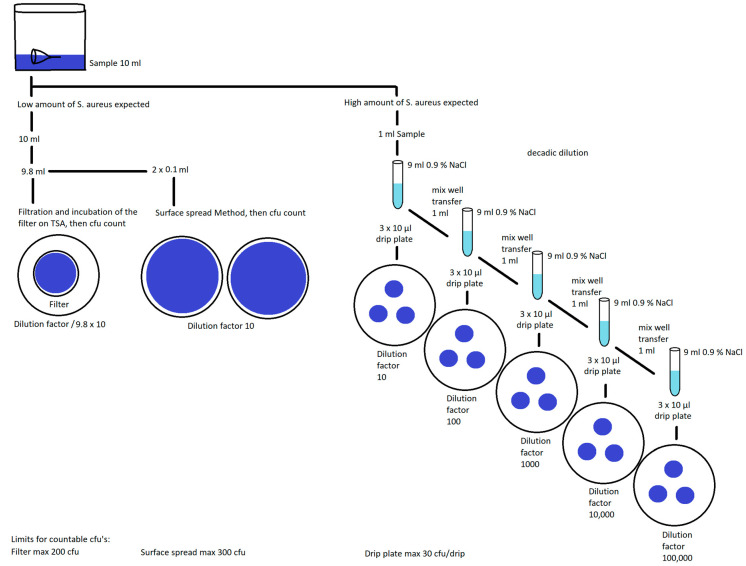
Diagram summarizing the experimental methods for viable bacterial cell abundance measurement. Legend: After incubation, colony-forming units (CFU) were measured. If there was a low amount of *S. aureus*, then the CFU was counted from surface plating or filtration on tryptic soy agar (TSA). If there was a high amount of *S. aureus*, then CFU was counted after applying the appropriate decadic dilution factor to obtain plates with 25 to 250 colonies.

After incubation, menstrual cups were cleaned using one of the four techniques described above. The number of *S. aureus* CFU still present after cleaning was determined with a stomacher^®^ bag test. Briefly, each cup was placed in a sterile stomacher^®^ bag (Stomacher 400 Circulator Lab Paddle Blender BA 0721 Homogenisator 400 mL, Seward, West Sussex, UK) containing 10 mL sterile NaCl-Tween (sodium chloride, p.a., Carl Roth, 3957.1). A decadic dilution series was performed in volumes of 1 mL of the sample in the stomacher^®^ bag plus 9 mL 0.9% sterile NaCl, at each step vortexed for 30 s and then shaken by hand for 20 s. Agar plates (Tryptic-Soy-Agar, Oxoid, PO5012A) were inoculated with 10 µL from each dilution in triplicate by the drop plate method, and plates were incubated at 37 °C for 12 h. After growth, plates with 25–250 colonies were used for counting, and CFU were determined applying the appropriate dilution factor to the number of colonies counted [19,20]. For samples with bacterial abundances below detection using the drop plate method, surface plating was performed to assess bacterial loads. Surface plating was conducted by spreading 100 µL of the stomacher^®^ bag solution on an agar plate. If no bacteria were recovered, then the entire volume of each stomacher^®^ bag was filtered through a 0.45 µm filter (EZ-Pak Filters, 47 mm white gridded, Millipore Sigma, Burlington, MA, USA), using a filter funnel and vacuum system. The cup and the stomacher^®^ bag were rinsed again with sterile NaCl (20–80 mL) to ensure that no bacteria remained. Filters were then placed directly on the tryptic soy agar plates and incubated for 12 h at 37 °C prior to colony counting (Figure 3).

### 2.5. Viable Bacterial Cell Abundance on Menstrual Cup Sections 

To determine the effect of the raised indentations (lettering) on bacterial growth and removal, viable bacterial counts were determined on 2 × 2 cm (4 cm^2^) pieces of the menstrual cup, with and without raised indentation from the lettering. Only the outer surface was evaluated as a prior test showed that the inner, smooth surface retained substantially fewer bacteria than the outer cup surface (data not shown). Two pieces, each with and without the raised indentation from the lettering (four pieces in total), were cut out of a complete cup. The outer surface of each piece of cup was coated with human blood with a cotton swab and then dried, as described above. Individual pieces of cup were placed in 6-micro-well plates (Sarstedt, 833.920) and covered with TSB medium (1.8 mL/well). After 12 h of incubation at 37 °C ± 1 °C (Memmert^®^ incubator), the pieces of cup were cleaned using four different cleaning methods similar to the above, but modified for small silicone pieces. Specifically, each piece of cup was rubbed between the fingers under running water for 4 s. In two cleaning scenarios, after handwashing the cup pieces, they were dropped in the ceramic mug, boiling water poured over it and left to steep for 5 min. CFU were counted as described for the complete cups, applying the stomacher^®^ test method followed by the drop plate, surface plating and, finally, the filter method.

### 2.6. Statistical Analysis

Statistical analyses were performed using the software package Stata/SE 15 (College Station, TX, USA). The statistical significance of differences in bacterial counts for each cleaning method compared to the reference (without cleaning) was determined using non-parametric testing. Because of the multiple paired comparisons between cleaning methods, we applied Dunn’s multiple comparison test with the Benjamini–Hochberg adjustment. For comparison of bacterial recovery from pieces with and without lettering, we applied the non-parametric Wilcoxon rank sum test as each of these were independent tests. For comparison of cleaning methods for pieces with and without lettering, Dunn’s test was applied, and *p*-values were adjusted for multiple comparisons.

## 3. Results

### 3.1. Impact of Different Cleaning Methods on Recovery of S. aureus from Entire Menstrual Cups

Without any cleaning (reference), there were on average 2.075 × 10^8^ CFU of *S. aureus* recovered per cup (Table 1, Method 0). With each successively stringent cleaning method, there was a 2- to 3-log reduction in recovery of CFU per cup (Table 1). Statistically significant (adjusted *p* < 0.05) reductions were observed for Method 3 (rubbing with cold tap water for 30 s followed by steeping the cup in boiling water in a mug for 5 min) and Method 4 (rubbing with soap and cold water for 30 s followed by immersion in boiling water in a mug for 5 min), resulting in recovery of an average of 14 (Method 3) and 0 CFU (Method 4) per cup.

### 3.2. Difference in Recovery of S. aureus from Surfaces of Menstrual Cup Pieces with and without Lettering, and Impact of Cleaning Methods

Across all test scenarios, the CFU recovered were higher for cup pieces with lettering than without lettering, although this did not reach statistical significance in any comparison (Table 2). Similar to the results with the whole cup pieces, each successive cleaning method led to multiple order-of-magnitude reductions in CFU recovered, with the greatest reduction observed when cup pieces were steeped in boiling water for 5 min.

## 4. Discussion

Evidence-informed cleaning recommendations are required to minimize the risk of menstrual cup-associated infection. A recent white paper report by PATH, a global public health non-profit organization, summarized qualitative analyses of menstrual cup products and cleaning instructions, and suggested a need for more evidence and consensus on effective cup cleaning to inform users and bolster support for the recommended cleaning practices [13]. Boiling for sterilization purposes is often recommended by manufacturers, but not all cup users have the ability to clean their menstrual cups by boiling them in a pot. In particular, the lack of privacy has been often mentioned by cup users as a limitation for this boiling approach [5]. Pouring boiled water over the cup and steeping instead of continuously boiling it in a pot avoids the problem of access to extended use of a stove or other heating mechanism. This method is more convenient and therefore likely more adoptable for many cup users. We therefore sought to test the effectiveness of steeping as a sterilization procedure in combination with other common and easy methods used to clean menstrual cups.

In our study, after seeding cups with a high bacterial load of *S. aureus*, we found that cleaning cups with soap and water and then steeping with boiled water for 5 min effectively eliminated bacterial recovery. The use of soap and boiling water reduced bacterial recovery to zero, though this was not significantly different to recovery from cups that were only cleaned through steeping in boiled water for 5 min without soap. However, while cleaning cups with soap and then steeping in boiled water had the greatest reduction in bacteria, using soap to wash the cups has not been recommended because soap can irritate the vagina. If soap can be adequately removed prior to use, this is the most effective sterilization method tested. Future observational studies of women using menstrual cups should assess actual cleaning practices, and whether these cleaning practices are associated with differences in vaginal microflora or vaginal symptoms.

Three previous in vitro studies have compared bacterial growth in menstrual products [21,22,23]. Nonfoux et al. suggested that menstrual cups could have a similar risk as tampons in causing mTSS, based on a laboratory experiment of *S. aureus* growth and TSST-1 production using the tampon sac method [22]. However, the authors acknowledged that the increased growth of *S. aureus* and TSST-1 production associated with cups in their study may have been a result of their experimental procedures, and could reflect the greater volume of air inserted into the experimental apparatus with cups relative to tampons, due to their shape. Thus, the Nonfoux et al. study does not fully quantitate the impact of menstrual cups on *S. aureus* growth and TSST-1 production in comparison to tampons. Schlievert performed a similar analysis of *S. aureus* growth with three methods: stationary incubation, incubation with shaking, or the tampon sac method (designed to mimic the vagina) [23]. In the tampon sac method, a bag (dialysis tubing) containing the period product was filled with growth medium and an *S. aureus* inoculum. Schlievert found no effect on *S. aureus* growth or production of TSST-1 on the menstrual cup pieces (2.5 cm^2^) as compared to controls without menstrual cup pieces. Similarly, in an older study, Tierno et al. found no production of TSST-1 on menstrual cups, though the Tassaway cups used were non-absorbent elastic polymer rather than silicone [21]. 

While these studies reflect the relevance of examining the association between menstrual cups and *S. aureus* growth, none systematically evaluated the effects of cleaning on bacterial recovery, which was the goal of our study. There are only a few studies that have examined how cup users clean their cups. Some data relate to cleaning the cup during a period (i.e., relating to reinsertion), while other data relate to cleaning the cup between periods. In South Africa, 93% of the cup users reported emptying and cleaning their menstrual cups at home with tap water when at home, though this decreased to 32–44% of women when having to empty outside the home [24]. In situations where there is no water, many cup users prefer to keep the cup in situ the whole day [25,26]. Systematic studies are needed to assess how women clean menstrual cups between reinsertions during their period, and between periods, and how this may relate to potential infection risk. Despite these knowledge gaps and variability in menstrual cup cleaning practices, in a systematic review with the meta-analysis by Van Eijk et al. [5], cases of mTSS in connection with menstrual cup use were rare, with only five reported cases in total through the period of review (up to May 2019). In some of these TSS cases, correlation with the cup usage was not proven, with women having other associated mTSS risk factors reported. Furthermore, research into the impact of menstrual cups on the vaginal microflora is limited, though a study by North et al. found no change over time [27]. Among 406 U.S. women, representing 4750 total days of menstrual cup use examined after 1, 2 and 3 months, there was no change in vaginal pH and vaginal bacterial cultivation detected no change in the rate of colonization with *S. aureus*, *G. vaginalis* or *Bacteroides* spp. In addition, the abundance of *Lactobacillus* spp. was maintained at normal levels. Among 604 Kenyan schoolgirls aged 14–16 years using menstrual cups over a median of 10.9 months, the *S. aureus* vaginal colonization prevalence did not statistically or meaningfully differ by MHM sanitary product/material, and was 9.4% among cups users versus 10.7% for pads and 10.5% controls [28]. TSST-forming bacteria were detected in two girls in the pad group, but none in the cup or control. In this small population, no adverse events or TSS were detected. Although these studies and post-marketing surveillance indicate menstrual cups are safe among women currently using them, it is necessary to address the knowledge gaps related to cleaning with the rapidly growing population of menstrual cup users, and an increasing number of brands becoming widely available.

### Limitations

The strengths of this in vitro study include novel assessment of steeping as a cleaning technique, and controlled conditions of biofilm formation and cleaning techniques, allowing replicability across labs and menstrual cups. However, population-based studies are needed to encompass the variability across individuals and how this is related to infection risk. For example, important mediating factors may include whether users have *S. aureus* and if it is TSST-1 producing; initial/first use cleaning, how long users wear their cup, cup characteristics (e.g., size, shape, firmness), how users clean the cup during a cycle, dropping or other adverse events, storage conditions and hand washing; and pathological conditions that may impair or make menstrual cup use more difficult [29]. We created ‘worst-case scenario’ conditions that favored *Staphylococcus* growth but which are unlikely in vivo. Different bacteria strains (e.g., *Lactobacillus*) can inhibit each other in the vagina, a mechanism that helps the host suppress growth of pathogenic bacteria [30]. The use of only one *S. aureus* strain means that there was no competition between different bacterial strains for nutrients and cell adhesion, further contributing to the ‘worst-case’ scenario we constructed in these experiments. While the comparison between cleaning methods and surface type (with and without lettering) indicates that some cleaning methods are more effective, the concentration of *S. aureus* grown in these experiments cannot be ‘scaled-up’ to an entire cup due to (1) the complete cup has a different surface structure; and (2) we used a shorter cleaning time (4 s) for the cup pieces as in reality one would not spend 30 s cleaning a 4 cm^2^ area. We conducted our study with liquid soap, as with bar soap it would have been more difficult to control the amount of soap used. However, this could be a limitation as many menstrual cup users in LMIC use bar soap. We did not test the fourth cleaning technique on the cup pieces, and this should be incorporated in future studies. Lastly, the effectiveness of the cleaning methods needs to be verified across different brands of menstrual cups, which have a varying texture, size, shape, and firmness, which could affect usage and safety [31].

## 5. Conclusions

In conclusion, we conducted an in vitro study to systematically evaluate the different cleaning methods for removing *S. aureus* from silicone menstrual cups. We observed that cleaning with tap water and soap and steeping in boiled water for 5 min was most effective at reducing viable bacteria, but that cleaning with tap water and steeping in boiled water for 5 min was not statistically significantly different. These results present an advancement in the knowledge of menstrual cups between-period cleaning recommendations, suggesting that the logistical challenges of continuous boiling may be eliminated with steeping. We advise that menstrual cup manufacturers, menstrual health programmers and donors advocate for and conduct studies across an increasing variety of cup types, along with evaluation in vivo, as understanding the real-world conditions would be of value.

## Figures and Tables

**Table 1:** Results of bacterial recovery under various cleaning methods for whole cups.

Test	Drop Plate Method (CFU/10 μL)		Surface Plating (CFU/ 100 μL)	Filter (CFU/ 9.8 μL)	Dilution Factor	Extraction Volume	CFU/Cup	Mean CFU/Cup	Dunn’s Multiple Test Adjusted *p*-Value for Each Method Compared to Reference
	1	2							
Method 0: Without cleaning (Reference)									
1	199	142			1000	100	1.705 × 10^8^		
2	233	217			1000	100	2.250 × 10^8^		
3	219	235			1000	100	2.270 × 10^8^	2.075 × 10^8^	
Method 1: Rubbed with cold tap water for 30 s									
1	88	87			1	100	8.75 × 10^4^		
2	144	144			1	100	1.44 × 10^5^		
3	12	12			10	100	1.20 × 10^5^	1.17 × 10^5^	0.205
Method 2: Rubbed with soap and cold tap water for 30 s									
1	87	112			0.5	100	9.95 × 10^3^		
2	70	62			0.5	100	6.60 × 10^3^		
3	84	56			0.5	100	7.00 × 10^3^	7.90 × 10^3^	0.099
Method 3: Rubbed with cold tap water for 30 s; steeped in boiling water and left for 5 min in mug with saucer cover									
1	<	<	<	24	1	99.8	24		
2	<	<	<	3	1	99.8	3		
3	<	<	<	11	1	99.8	11	14	0.022
Method 4: Rubbed with soap and cold tap water for 30 s; steeped in boiling water and left for 5 min in mug with saucer cover									
1	<	<	<	0	1	100	0		
2	<	<	<	0	1	100	0		
3	<	<	<	0	1	100	0	0	0.005

CFU = colony-forming units; ≤ below limit of detection.

**Table 2:** Results of bacterial recovery under various washing conditions for 2 × 2 cm cup pieces to examine the effect of external cup lettering on bacterial recovery.

	Drop Plate Method (CFU/ 10 μL)			Surface Plating (CFU/ 100 μL)	Filter (CFU/ 9.8 μL)	Dilution Factor	CFU/ Cup Piece	Mean	Within Washing Wilcoxon Rank Sum *p*-Value for Comparison of with Lettering vs. without Lettering	Between Washing Dunn’s Multiple Test Adjusted *p*-Value For Comparison of Each Cleaning Method vs. Reference
	1	2	3							
Method 0: Without cleaning (Reference)										
Without lettering	19	22	13	>	>	100	1.80 × 10^7^			
Without lettering	9	10	10	>	>	100	9.67 × 10^6^	1.38 × 10^7^		
With lettering	16	11	13	>	>	10,000	1.33 × 10^8^			
With lettering	19	15	18	>	>	10,000	1.73 × 10^8^	1.53 × 10^8^	0.121	
Method 1: Rubbed with cold tap water only for 4 s										
Without lettering	27	20	38	>	>	100	2.83 × 10^6^			
Without lettering	24	24	29	>	>	100	2.57 × 10^6^	2.70 × 10^6^		0.249
With lettering	4	7	14	>	>	1000	8.33 × 10^6^			
With lettering	11	7	9	>	>	100	9.00 × 10^5^	4.62 × 10^6^	>0.999	0.206
Method 2: Rubbed with soap and cold tap water for 4 s										
Without lettering	<	<	<	28	>	1	2.80 × 10^3^			
Without lettering	<	<	<	19	>	1	1.90 × 10^3^	2.35 × 10^3^		0.103
With lettering	13	12	13	>	>	1	1.27 × 10^4^			
With lettering	12	14	12	>	>	1	1.27 × 10^4^	1.27 × 10^4^	0.103	0.151
Method 3: Rubbed with cold tap water only for 4 s; steeped in boiled water and left for 5 min in mug with saucer cover										
Without lettering	<	<	<	<	13	1	13			
Without lettering	<	<	<	<	7	1	7	10		0.043
With lettering	<	<	<	<	149	1	152			
With lettering	<	<	<	<	123	1	125	139	0.121	0.041

CFU = colony-forming unit; ≤ below detection; ≥ above detection.

## Data Availability

All the raw data are included in Table 1 and Table 2.

## References

[1] Sommer M., Caruso B.A., Sahin M., Calderon T., Cavill S., Mahon T., Phillips-Howard P.A. (2016). A time for global action: Addressing girls’ menstrual hygiene management needs in schools. PLoS Med..

[2] Sommer M., Chandraratna S., Cavill S., Mahon T., Phillips-Howard P.A. (2016). Managing menstruation in the workplace: An overlooked issue in low-and middle-income countries. Int. J. Equity Health.

[3] Van Eijk A.M., Sivakami M., Thakka M.B., Bauman A., Laserson K.F., Coates S., Phillips-Howard P.A. (2016). Menstrual hygiene management among adolescent girls in India: A systematic review and meta-analysis. BMJ Open.

[4] Hait A., Powers S.E. (2019). The value of reusable feminine hygiene products evaluated by comparative environmental life cycle assessment. Resour. Conserv. Recycl..

[5] Van Eijk A.M., Zulaika G., Lenchner M., Mason L., Sivakami M., Nyothach E., Unger H., Laserson K., Phillips-Howard P.A. (2019). Menstrual cup use, leakage, acceptability, safety, and availability: A systematic review and meta-analysis. Lancet Public Health.

[6] Beksinska M., Nkosi P., Zulu B., Smit J. (2021). Acceptability of the menstrual cup among students in further education institutions in KwaZulu-Natal, South Africa. Eur. J. Contracept. Reprod. Health Care.

[7] Mason L., Nyothach E., van Eijk A.M., Obor D., Alexander K.T., Ngere I., Laserson K., Phillips-Howard P. (2019). Comparing use and acceptability of menstrual cup and sanitary pads by schoolgirls in rural Western Kenya. Int. J. Reprod. Contracept. Obstet. Gynecol..

[8] Schlievert P.M., Nemeth K.A., Davis C.C., Peterson M.L., Jones B.E. (2010). *Staphylococcus aureus* exotoxins are present in vivo in tampons. Clin. Vaccine Immunol..

[9] Schlievert P.M., Blomster D.A. (1983). Production of staphylococcal pyrogenic exotoxin type C: Influence of physical and chemical factors. J. Infect. Dis..

[10] Hajjeh R.A., Reingold A., Weil A., Shutt K., Schuchat A., Perkins B.A. (1999). Toxic shock syndrome in the United States: Surveillance update, 1979–1996. Emerg. Infect. Dis..

[11] WoMena (2021). FAQs: How Many Women Menstruate? Use Menstrual Cups? What Is The Environmental Impact?. https://womena.dk/how-many-women-menstruate/.

[12] Veeh R.H., Shirtliff M.E., Petik J.R., Flood J.A., Davis C.C., Seymour J.L., Hansmann M.A., Kerr K.M., Pasmore M.E., Costerton J.W. (2003). Detection of *Staphylococcus aureus* biofilm on tampons and menses components. J. Infect. Dis..

[13] (2021). PATH Menstrual Cup Cleaning Practices: A Mixed Methods Study of Published Instructions and Key Informant Interviews. https://path.azureedge.net/media/documents/Menstrual_Cup_Cleaning_Practices.pdf.

[14] African Population and Health Research Center (APHRC) (2010). Experiences and Problems with Menstruation among Poor Women and Schoolgirls in Nairobi, Kenya Policy No. Brief 20. https://www.susana.org/en/knowledge-hub/resources-and-publications/library/details/983.

[15] Tellier M., Hyttel M., Gad M. (2012). Assessing Acceptability and Hygienic Safety of Menstrual Cups as Menstrual Management Methods for Vulnerable Young Women in Uganda Red Cross Society’s Life Planning Skills Project. WoMena. https://womena.dk/publication-pilot-study-report-on-menstrual-cups-for-vulnerable-young-women-in-uganda/.

[16] Hyttel M., Thomsen C.F., Luff B., Storrusten H., Nyakato V.N., Tellier M. (2017). Drivers and challenges to use of menstrual cups among schoolgirls in rural Uganda: A qualitative study. Waterlines.

[17] Einhorn Landing Page. https://einhorn.my.

[18] Einhorn Frequently Asked Questions. https://einhorn.my/faq/.

[19] American Society for Testing and Material (ASTM) D5465-16 (2020). Standard Practices for Determining Microbial Colony Counts from Waters Analyzed by Plating Methods.

[20] Maturin L., Peeler J.T. BAM Chapter 3: Aerobic Plate Count. United States Food and Drug. https://www.fda.gov/food/laboratory-methods-food/bam-chapter-3-aerobic-plate-count.

[21] Tierno P.M., Hanna B.A. (1994). Propensity of tampons and barrier contraceptives to amplify *Staphylococus aureus* toxic shokc syndrome toxin-1. Infect. Dis. Obstet. Gynecol..

[22] Nonfoux L., Chiaruzzi M., Badiou C., Baude J., Tristan A., Thioulouse J., Muller D., Prigent-Combaret C., Lina G. (2018). Impact of currently marketed tampons and menstrual cups on *Staphylococcus aureus* growth and toxic shock syndrome toxin 1 production in vitro. Appl. Environ. Microbiol..

[23] Schlievert P.M. (2020). Effect of non-absorbent intravaginal menstrual/contraceptive products on *Staphylococcus aureus* and production of the superantigen TSST-1. Eur. J. Clin. Microbiol. Infect. Dis..

[24] Beksinska M.E., Smit J., Greener R., Todd C.S., Lee M.T., Maphumulo V., Hoffmann V. (2015). Acceptability and performance of the menstrual cup in South Africa: A randomized crossover trial comparing the menstrual cup to tampons or sanitary pads. J. Womens Health (Larchmt).

[25] African Population and Health Research Center (APHRC) (2010). Policy Brief 22: Use of Menstrual Cup by Adolescent Girls and Women: Potential Benefits and Key Challenges. https://www.susana.org/en/knowledge-hub/resources-and-publications/library/details/985.

[26] Mason L., Laserson K.F., Oruko K., Nyothach E., Alexander K., Odhiambo F., Eleveld A., Isiye E., Ngere I., Omoto J. (2015). Adolescent schoolgirls’ experiences of menstrual cups and pads in rural western Kenya: A qualitative study. Waterlines.

[27] North B.B., Oldham M.J. (2011). Preclinical, clinical, and over-the-counter postmarketing experience with a new vaginal cup: Menstrual collection. J. Womens Health (Larchmt).

[28] Juma J., Nyothach E., Laserson K.F., Oduor C., Arita L., Ouma C., Oruko K., Omoto J., Mason L., Alexander K.T. (2017). Examining the safety of menstrual cups among rural primary school girls in western Kenya: Observational studies nested in a randomised controlled feasibility study. BMJ Open.

[29] Motta T., Lagana A.S., Valenti G., La Rosa V.L., Noventa M., Vitagliano A., Noventa M., Vitagliano A., Chiofalo B., Rapisarda A.M. (2017). Differential diagnosis and management of abnormal uterine bleeding in adolescence. Minverva. Ginecol..

[30] Amabebe E., Anumba D.O.C. (2018). The vaginal microenvironment: The physiologic role of Lactobacilli. Front. Med. (Lausanne).

[31] Manley H., Hunt J.A., Santos L., Breedon P. (2021). Comparison between menstrual cups: First step to categorization and improved safety. Womens Health.

